# Long-term risk of incident thyroid cancer after COVID-19 in patients with thyroid disorder: a multicenter propensity score–matched cohort study

**DOI:** 10.3389/fendo.2026.1926365

**Published:** 2026-08-11

**Authors:** Ming Yew, Wei-Ting Wang, Ping-Hsun Feng

**Affiliations:** 1 Department of Anesthesiology, Chi Mei Medical Center, Tainan, Taiwan; 2 Department of Anesthesiology, E-Da Hospital, I-Shou University, Kaohsiung, Taiwan; 3 Department of Anesthesiology, Chi Mei Hospital, Liouying, Tainan, Taiwan

**Keywords:** COVID-19, propensity score matching, SARS-CoV-2, surveillance bias, thyroid cancer, thyroid disease

## Abstract

**Background:**

Prior studies examining the relationship between coronavirus disease 2019 (COVID-19) and thyroid cancer in general populations may be influenced by surveillance bias, complicating interpretation of their findings. To address this limitation, we evaluated the association between severe acute respiratory syndrome coronavirus 2 (SARS-CoV-2) infection and incident thyroid cancer specifically among adults with pre-existing thyroid disease.

**Methods:**

We used the TriNetX Global Collaborative Network to conduct a propensity score–matched cohort study of adults aged ≥18 years with thyroid disorders between 2020 and 2022. Patients with documented COVID-19 were compared with matched controls without recorded SARS-CoV-2 infection. A 24-month landmark design excluded cancers diagnosed during early post-infectious evaluation. The primary outcome was incident thyroid cancer, estimated using Cox proportional hazards models, and all-cause mortality was assessed as a secondary outcome. Follicular cysts of skin served as a negative-control outcome. E-values were calculated to assess the robustness of the observed association to potential unmeasured confounding. Sensitivity, subgroup, alternative landmark, hospitalization-based severity-stratified, competing-risk, and secondary generalizability analyses were performed.

**Results:**

After matching, 603,364 patients were included in each cohort. COVID-19 was associated with a higher risk of incident thyroid cancer (hazard ratio [HR], 1.61; 95% confidence interval [CI], 1.49–1.75; E-value, 2.60). The association persisted across sensitivity analyses (HRs, 1.70–1.95), sex and age strata, baseline thyroid-disease subtypes, hospitalization status, and 1- to 3-year landmark analyses. Similar findings were observed in a secondary cohort of adults with type 2 diabetes and no prior thyroid disease (HR, 1.78; 95% CI, 1.53–2.07). All-cause mortality was higher in the COVID-19 cohort compared with controls (HR, 1.70; 95% CI, 1.66–1.73). The negative-control outcome showed no increase in cumulative incidence (risk ratio, 0.95) despite a modestly higher hazard (HR, 1.16; 95% CI, 1.13–1.19).

**Conclusion:**

Among adults with pre-existing thyroid disease, COVID-19 was associated with a delayed increase in incident thyroid cancer. This association was not readily explained by measured healthcare utilization alone, but residual surveillance bias cannot be excluded. These findings represent an epidemiologic signal rather than proof of causation and do not support routine thyroid cancer screening based solely on prior COVID-19.

## 1 Background

Thyroid cancer is the most common endocrine malignancy, and its reported incidence has increased over recent decades, largely because of incidental detection of small papillary cancers during imaging performed for unrelated indications (1–4). Established risk factors include ionizing radiation and obesity (5–7), but pre-existing thyroid disease is also clinically relevant. Compared with individuals without thyroid disease, patients with Hashimoto’s thyroiditis, Graves’ disease, or nodular goiter have been reported to have a higher risk of thyroid cancer (8–12). For example, a population-based cohort study reported thyroid cancer incidence rates of 353 and 22 cases per 100,000 person-years among patients with thyroiditis and matched individuals without thyroiditis, respectively (13). However, the magnitude of risk varies considerably across different thyroid disorders and may be influenced by diagnostic intensity. These conditions may contribute through chronic inflammation, autoimmune dysregulation, or structural glandular changes that predispose to malignant transformation (14–16). Patients with these disorders also undergo periodic biochemical assessment and structural imaging, making them both a high-risk population and a group with relatively consistent opportunities for cancer detection.

Whether severe acute respiratory syndrome coronavirus 2 (SARS-CoV-2) infection further increases thyroid cancer risk in this setting remains unclear (17–19). The thyroid gland is biologically plausible as a target organ because angiotensin-converting enzyme 2 (ACE2) is expressed in thyroid follicular cells, and SARS-CoV-2 ribonucleic acid (RNA) has been detected in thyroid tissue (20–22). Infection-related cytokine activation, oxidative stress, immune dysregulation, and perturbation of tumor-suppressor pathways have been proposed as mechanisms that could contribute to carcinogenesis (23–25). Clinically, coronavirus disease 2019 (COVID-19) has been associated with subacute thyroiditis, transient thyrotoxicosis, and incident autoimmune thyroid disease (26–30). However, most studies have focused on short-term thyroid dysfunction rather than delayed thyroid neoplasia.

A recent TriNetX-based multicenter study reported a modest association between COVID-19 and incident thyroid cancer in the general adult population, with stronger estimates among individuals who developed post-infectious thyroid dysfunction (31). However, several methodological issues complicate interpretation, including limited propensity score adjustment, lack of a formal landmark design, stratification by a post-exposure intermediate variable, and absence of negative-control or healthcare-utilization analyses. More broadly, studies in the general population remain susceptible to surveillance bias, because patients recovering from COVID-19 may have more frequent healthcare encounters, thereby increasing the incidental detection of small, indolent thyroid cancers.

Building on these considerations, the present study was specifically designed to address these methodological limitations by restricting the analysis to adults with pre-existing thyroid disease, thereby partly reducing surveillance bias by focusing on patients already engaged in thyroid surveillance while addressing a clinically actionable high-risk population. This approach also helps ensure more consistent follow-up and detection practices across study groups. Nevertheless, even within this enriched population, careful study design is needed to distinguish a delayed association with thyroid cancer from early diagnostic activity after infection. Using the TriNetX Global Collaborative Network, we therefore evaluated the long-term risk of incident thyroid cancer after SARS-CoV-2 infection among adults with pre-existing thyroid disease, incorporating propensity score matching and a 2-year landmark analysis to mitigate bias and assess temporal associations.

## 2 Methods

### 2.1 Study design and data source

We conducted a retrospective, propensity score–matched cohort study using the TriNetX Global Collaborative Network, a federated platform aggregating de-identified electronic health records from 179 participating healthcare organizations across multiple countries. The TriNetX platform has been widely used in peer-reviewed observational studies across diverse clinical disciplines (32–34). The network provides patient-level diagnoses, procedures, medications, and laboratory values mapped to standardized terminologies (International Classification of Diseases, Tenth Revision, Clinical Modification [ICD-10-CM]; Logical Observation Identifiers Names and Codes [LOINC]; RxNorm; and TriNetX-curated codes). Because the platform returns only aggregated, de-identified counts and statistics, the study protocol was approved by the Institutional Review Board of Chi Mei Medical Center, and the requirement for informed consent was waived. The study was conducted in accordance with the principles of the Declaration of Helsinki.

### 2.2 Study population and exposure definition

Eligible patients were adults aged 18 years or older who had a documented disorder of the thyroid gland, defined using ICD-10-CM codes E00–E07, between January 1, 2020 and June 30, 2022. Patients were assigned to the COVID-19 cohort if they had either a diagnosis code for COVID-19 (U07.1) or a positive SARS-CoV-2 RNA laboratory result (TNX:9088) during the ascertainment period. Patients were assigned to the control cohort if they had a documented thyroid disorder during the same period but no record of either COVID-19 diagnosis or SARS-CoV-2 RNA positivity.

The index date was defined as the first date on which a patient satisfied the qualifying cohort criteria. Patients were required to have at least one recorded healthcare encounter during the 5-year baseline period before the index date to ensure adequate baseline information. A 24-month landmark design was prespecified as a pragmatic, study-specific interval rather than selected from a disease-specific guideline or a previously validated threshold. This interval was chosen to create a conservative separation from early post-infectious diagnostic activity, thereby allowing the analysis to focus on delayed incident thyroid cancer and reduce early ascertainment effects (35). Patients with malignant neoplasm of the thyroid gland (C73) before or within 24 months after the index date, as well as those who died during the landmark period, were excluded. Follow-up began at the 24-month landmark and continued until thyroid cancer diagnosis, death, the last available record, or 6 years after the index date, whichever occurred first.

### 2.3 Exclusion criteria

Additional baseline exclusions were applied to reduce confounding from conditions strongly related to cancer risk, thyroid malignancy detection, or competing clinical trajectories. Patients were excluded if they had advanced chronic kidney disease or renal replacement status, including chronic kidney disease stage 4 or 5, end-stage renal disease, or dependence on renal dialysis; type 1 diabetes mellitus; or gestational diabetes mellitus. We also excluded patients with systemic connective tissue disorders, prior malignant neoplasms of the head and neck region, malignant neoplasms of the lip, oral cavity, pharynx, nasal cavity, middle ear, accessory sinuses, or larynx, benign neoplasm of the thyroid gland, carcinoma *in situ* of the thyroid or other endocrine glands, personal history of irradiation, or family history of primary malignant neoplasm. All inclusion and exclusion criteria were applied symmetrically to the COVID-19 and control cohorts, except for the SARS-CoV-2 exposure definition. All diagnostic criteria and code mappings used to operationalize each variable are listed in **Supplementary Table 1**.

### 2.4 Propensity score matching

To reduce baseline differences between cohorts, 1:1 propensity score matching was performed using a greedy nearest-neighbor algorithm with a caliper of 0.1 pooled standard deviations of the propensity score logit. The matching model incorporated demographic characteristics, race, body mass index, cardiovascular, renal, hepatic, respiratory, and metabolic comorbidities, malignancy, smoking- and alcohol-related diagnoses, malnutrition, COVID-19 vaccination status, laboratory markers, thyroid disease subtypes (e.g., hypothyroidism, hyperthyroidism), thyroid-related medications (e.g., thyroid hormone replacement), other relevant medication classes, and thyroid-specific testing and imaging procedures; all covariates are enumerated in **Supplementary Table 2**. Covariate balance was assessed using standardized mean differences (SMDs), with values below 0.10 considered acceptable. Propensity score density plots were used to visually confirm distributional overlap before and after matching.

### 2.5 Outcomes

The primary outcome was incident thyroid cancer, defined by malignant neoplasm of the thyroid gland (C73), while all-cause mortality was assessed as a secondary outcome. To better understand potential detection bias, we also included hypothyroidism (E03), nontoxic goiter (E04), and hyperthyroidism (E05) as thyroid-related follow-up indicators. Because these diagnoses may represent either pre-existing thyroid disease requiring ongoing care or newly recorded thyroid dysfunction during follow-up, they were not interpreted as incident thyroid outcomes but were instead used to reflect the extent of subsequent thyroid-related clinical attention and diagnostic recording in each cohort. Follicular cysts of skin and subcutaneous tissue (L72) served as a negative-control outcome because they represent a benign dermatologic condition with no plausible causal relationship to SARS-CoV-2 infection or thyroid carcinogenesis, while their documentation may nevertheless be influenced by routine healthcare contact and diagnostic recording. They were therefore selected according to general negative-control methodology, rather than on the basis of a prior COVID-19 or thyroid cancer publication, to help identify nonspecific healthcare-utilization or ascertainment bias (36). The occurrence of any healthcare visit was assessed as a general healthcare-utilization marker, and the per-patient frequency of thyroid-related diagnostic records was summarized to evaluate potential differential follow-up intensity between cohorts.

### 2.6 Sensitivity and subgroup analyses

Seven sensitivity analyses were conducted to evaluate whether the observed association was robust to potential surveillance- and survival-related artifacts. Model I restricted the cohort to patients who survived throughout follow-up. Model II included only patients with at least one healthcare encounter during follow-up, thereby reducing bias from differential retention in the electronic health record system. Model III was limited to patients who had thyroid-related testing or evaluation during follow-up. Model IV further restricted the analysis to patients with an abnormal thyroid-stimulating hormone (TSH) value (<0.4 or >4 mIU/L) during follow-up. Model V restricted the cohort to patients with baseline hypothyroidism, Model VI to those with baseline hyperthyroidism, and Model VII to those with baseline goiter. Additional subgroup analyses were performed by sex and age group (18–65 versus >65 years). Interaction was assessed by comparing stratum-specific log hazard ratios, with corresponding P-values for interaction reported.

### 2.7 Landmark sweep, severity-stratified, and descriptive competing-risk analyses

To evaluate the temporal stability of the association and the influence of different event-free periods, additional landmark analyses were performed using 1-year and 3-year landmarks, with the 2-year landmark serving as the primary analytic framework. These analyses assessed whether the association between COVID-19 and subsequent thyroid cancer persisted when the start of outcome ascertainment was shifted earlier or later after the index date and whether the findings depended on the prespecified 2-year interval. We also conducted a hospitalization-based severity-stratified analysis comparing non-hospitalized and hospitalized COVID-19 patients with separately matched controls to assess whether thyroid cancer risk differed by hospitalization status and whether greater illness severity or healthcare intensity was associated with a stronger signal. This analysis also explored potential detection bias related to increased medical evaluation in hospitalized patients and was not interpreted as a formal dose–response analysis. Finally, a descriptive competing-risk analysis was conducted among unmatched patients using the Aalen–Johansen estimator to display the cumulative incidence of thyroid cancer while modeling death as a competing event, without formal statistical comparison between groups.

### 2.8 Additional analysis in patients with type 2 diabetes and no prior thyroid disease

To assess the consistency and generalizability of the findings beyond the primary thyroid-disease cohort, we conducted a secondary analysis in a distinct clinical population comprising adults with type 2 diabetes mellitus and no documented pre-existing thyroid disease. This analysis applied the same SARS-CoV-2 exposure definition, 2-year landmark design, propensity score matching procedure, and outcome and control definitions used in the primary analysis. Because this analysis was performed within the same data network, it was considered a supplementary generalizability analysis rather than a formal external validation.

### 2.9 Statistical analysis

Baseline characteristics were summarized as means with standard deviations for continuous variables and as frequencies with percentages for categorical variables. In the matched cohorts, risk ratios (RRs) were derived from the proportion of patients experiencing each outcome, and time-to-event associations were estimated using Cox proportional hazards models, with hazard ratios (HRs) and 95% confidence intervals (CIs) reported alongside log-rank tests. The proportional hazards assumption was evaluated using the proportionality test implemented in the TriNetX platform. For the primary outcome, E-values quantify the robustness of an observed association to potential unmeasured confounding. They estimate the minimum strength of association, on the risk-ratio scale, that an unmeasured confounder would need to have with both the exposure and outcome to fully explain the observed estimate and its lower confidence bound. Missing data were not imputed; all analyses used available data within the platform. Two-sided p-values below 0.05 were considered statistically significant for the primary outcome. Because the secondary, sensitivity, subgroup, landmark, hospitalization-based severity-stratified, healthcare-utilization, and competing-risk analyses were regarded as exploratory, no adjustment for multiple comparisons was applied.

### 2.10 Use of artificial intelligence tools

During manuscript preparation, the authors used a large language model–based assistant to support language editing and to check internal consistency between the text and the tables. The tool was not used to generate or interpret study data. The authors reviewed and verified all content and take full responsibility for the integrity and accuracy of the work. No large language model meets authorship criteria, and none is listed as an author.

## 3 Results

### 3.1 Cohort assembly and covariate balance

**Figure 1:**
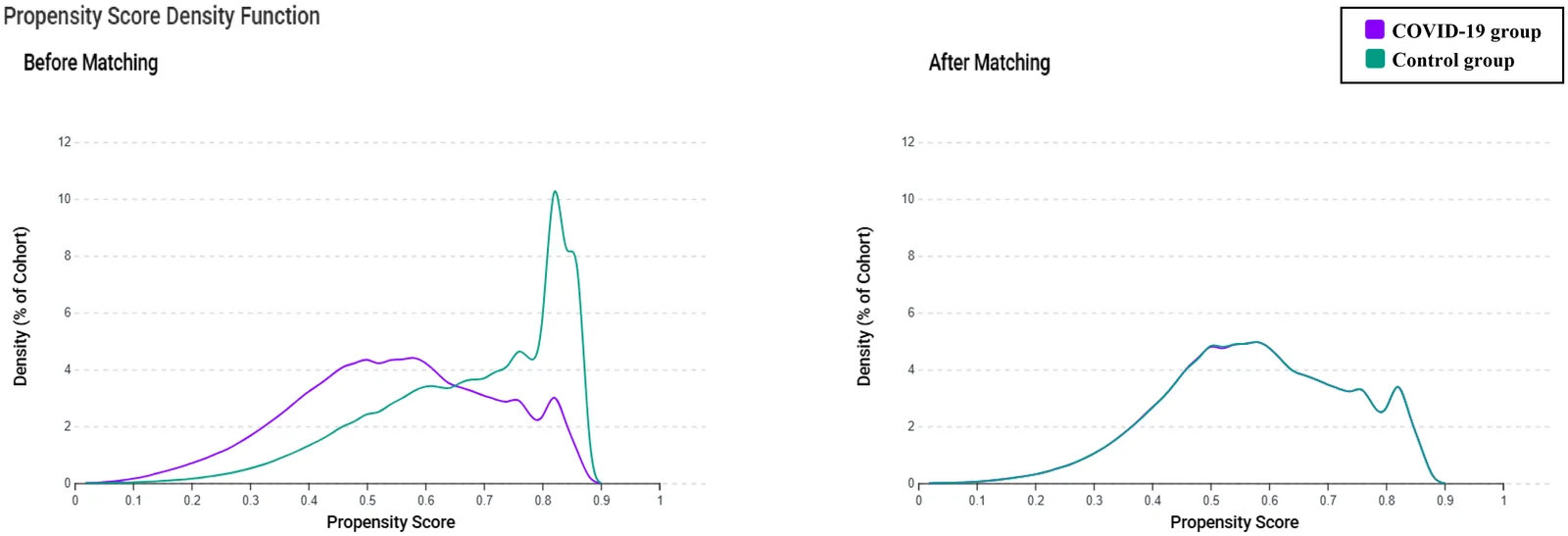
Propensity score distributions before and after matching. Propensity score density plots comparing adults with pre-existing thyroid disease and documented COVID-19 with matched controls without documented SARS-CoV-2 infection before and after 1:1 propensity score matching. Before matching, the two cohorts showed partial separation in propensity score distributions. After matching, the distributions demonstrated substantial overlap, indicating improved covariate balance between groups.

The analysis comprised 702,555 individuals with pre-existing thyroid disease who had documented COVID-19 and 1,255,089 individuals with thyroid disease but no evidence of SARS-CoV-2 infection (**Table 1**). Following implementation of a 24-month landmark approach and 1:1 propensity score matching, both groups were reduced to 603,364 participants. Prior to matching, those with COVID-19 exhibited a greater prevalence of obesity, hypothyroidism, hypertension, dyslipidaemia, diabetes, cardiovascular conditions, medication use, and thyroid-related testing. After matching, baseline characteristics were well aligned between groups, with all standardized mean differences falling below 0.10. This balance was further supported by propensity score density plots (**Figure 1**), which demonstrated substantial overlap between the cohorts.

**Table 1:** Baseline characteristics of patients with thyroid disease with and without COVID-19 before and after propensity score matching.

Variables	Before matching			After matching			
	COVID-19 group (n = 702,555)	Control group (n = 1,255,089)	SMD		COVID-19 group (n = 603,364)	Control group (n = 603,364)	SMD
Patient characteristics							
Age at index (years)	59.2 ± 17.8	56.9 ± 18.1	0.132		58.5 ± 18.1	59.1 ± 17.5	0.031
Female	532758 (75.8)	971731 (77.4)	0.038		461530 (76.5)	459388 (76.1)	0.008
BMI>30 kg/m^2^	282719 (40.2)	324330 (25.8)	0.310		222691 (36.9)	227656 (37.7)	0.017
White	521324 (74.2)	824287 (65.7)	0.187		441840 (73.2)	450268 (74.6)	0.032
Black or African American	71448 (10.2)	100248 (8.0)	0.076		57825 (9.6)	57957 (9.6)	0.001
Asian	24037 (3.4)	63963 (5.1)	0.083		22609 (3.7)	22778 (3.8)	0.001
Comorbidities and health services							
Other hypothyroidism	376063 (53.5)	431973 (34.4)	0.392		304408 (50.5)	311778 (51.7)	0.024
Essential (primary) hypertension	332323 (47.3)	377067 (30.0)	0.360		259548 (43.0)	261939 (43.4)	0.008
Disorders of lipoprotein metabolism and other lipidemias	323265 (46.0)	389758 (31.1)	0.311		257642 (42.7)	261384 (43.3)	0.013
Neoplasms	197835 (28.2)	227553 (18.1)	0.239		153961 (25.5)	155108 (25.7)	0.004
Overweight and obesity	177250 (25.2)	168391 (13.4)	0.303		130598 (21.6)	128887 (21.4)	0.007
Diabetes mellitus	142445 (20.3)	154074 (12.3)	0.218		106350 (17.6)	106044 (17.6)	0.001
Nontoxic goiter	111687 (15.9)	131692 (10.5)	0.160		87711 (14.5)	89094 (14.8)	0.006
Ischemic heart diseases	103718 (14.8)	88354 (7.0)	0.250		69844 (11.6)	68036 (11.3)	0.009
Nicotine dependence	75408 (10.7)	63821 (5.1)	0.210		52493 (8.7)	50989 (8.5)	0.009
Cerebrovascular diseases	62390 (8.9)	52658 (4.2)	0.190		41943 (7.0)	40892 (6.8)	0.007
Diseases of liver	59847 (8.5)	52146 (4.2)	0.180		40994 (6.8)	39785 (6.6)	0.008
Other disorders of thyroid	53709 (7.6)	55070 (4.4)	0.137		40501 (6.7)	41268 (6.8)	0.005
Chronic kidney disease (CKD)	59722 (8.5)	49085 (3.9)	0.191		39643 (6.6)	38655 (6.4)	0.007
Thyrotoxicosis [hyperthyroidism]	46946 (6.7)	63648 (5.1)	0.069		37831 (6.3)	38406 (6.4)	0.004
Other chronic obstructive pulmonary disease	55540 (7.9)	39399 (3.1)	0.210		34000 (5.6)	32427 (5.4)	0.011
Heart failure	55215 (7.9)	35095 (2.8)	0.227		31901 (5.3)	29914 (5.0)	0.015
Alcohol related disorders	20909 (3.0)	13813 (1.1)	0.133		12645 (2.1)	12035 (2.0)	0.007
Malnutrition	12610 (1.8)	6140 (0.5)	0.123		6309 (1.0)	5611 (0.9)	0.012
Medications							
Glucocorticoids	339514 (48.3)	337149 (26.9)	0.454		259988 (43.1)	259972 (43.1)	0.000
Thyroid hormones	313096 (44.6)	397595 (31.7)	0.268		256248 (42.5)	264158 (43.8)	0.026
Antilipemic agents	234598 (33.4)	254879 (20.3)	0.299		178654 (29.6)	180265 (29.9)	0.006
COVID-19 Vaccine	105277 (15.0)	80595 (6.4)	0.280		61827 (10.2)	56004 (9.3)	0.033
Insulins and analogues	77507 (11.0)	57616 (4.6)	0.242		49843 (8.3)	47603 (7.9)	0.014
GLP-1 analogues	21742 (3.1)	19366 (1.5)	0.103		14973 (2.5)	14635 (2.4)	0.004
Antithyroid preparations	14918 (2.1)	26062 (2.1)	0.003		12776 (2.1)	13178 (2.2)	0.005
SGLT2 inhibitors	15052 (2.1)	14594 (1.2)	0.077		10597 (1.8)	10465 (1.7)	0.002
Laboratory data							
eGFR≥60 mL/min/1.73 m²	511805 (72.8)	611566 (48.7)	0.510		420285 (69.7)	425760 (70.6)	0.020
Hemoglobin ≥12 g/dL	511705 (72.8)	582819 (46.4)	0.559		416664 (69.1)	421782 (69.9)	0.018
Albumin ≥3.5 g/dL	480154 (68.3)	543917 (43.3)	0.520		388338 (64.4)	392082 (65.0)	0.013
HbA1c≥9%	26036 (3.7)	23220 (1.9)	0.113		18046 (3.0)	17600 (2.9)	0.004
TSH 0.4–4 m[IU]/L	394011 (56.1)	473318 (37.7)	0.375		320260 (53.1)	325778 (54.0)	0.018
Thyroid-related Procedure							
TSH	295226 (42.0)	305025 (24.3)	0.383		228352 (37.8)	228572 (37.9)	0.001
Diagnostic Ultrasound Procedures of the Head and Neck	76336 (10.9)	82077 (6.5)	0.154		58273 (9.7)	58381 (9.7)	0.001

Data are presented as number (%) or mean ± standard deviation. Propensity score matching was performed at a 1:1 ratio using a greedy nearest-neighbor algorithm with a caliper of 0.1 pooled standard deviation of the propensity score logit. Standardized mean differences <0.10 indicated acceptable covariate balance. BMI, body mass index; CKD, chronic kidney disease; COVID-19, coronavirus disease 2019; eGFR, estimated glomerular filtration rate; GLP-1, glucagon-like peptide-1; HbA1c, hemoglobin A1c; SGLT2, sodium-glucose cotransporter 2; SMD, standardized mean difference; TSH, thyroid-stimulating hormone.

### 3.2 Incident thyroid cancer, mortality, and follow-up diagnostic records

After the 24-month landmark, incident thyroid cancer occurred in 1,371 COVID-19 patients and 1,055 controls (0.23% vs 0.18%) (**Table 2**). COVID-19 was associated with higher risk (HR 1.61, 95% CI 1.49–1.75; log-rank p<0.001). The E-value was 2.60 for the point estimate and 2.34 for the lower confidence limit, indicating that an unmeasured confounder would require a moderate association with both COVID-19 and thyroid cancer to fully explain the observed estimate.

**Table 2:** Association of COVID-19 with incident thyroid cancer, mortality, and follow-up thyroid-related diagnostic records among patients with pre-existing thyroid disease.

Outcome	COVID-19 group (n=603,364)	Control group (n=603,364)	RR (95% CI)	HR (95% CI)	*p* value
	Events (%)	Events (%)			
Primary outcome					
Thyroid cancer	1,371 (0.23%)	1,055 (0.18%)	1.30 (1.20–1.41)	1.61 (1.49–1.75)	<0.001
Secondary outcome					
Mortality	22,134 (3.67%)	16,822 (2.79%)	1.32 (1.29–1.34)	1.70 (1.66–1.73)	<0.001
Follow-up thyroid-related diagnostic records					
Hypothyroidism	249,181 (41.30%)	287,809 (47.70%)	0.87 (0.86–0.87)	0.87 (0.87–0.88)	<0.001
Goiter	59,397 (9.84%)	74,498 (12.35%)	0.80 (0.79–0.81)	0.87 (0.86–0.88)	<0.001
Hyperthyroidism	25,912 (4.30%)	31,717 (5.26%)	0.82 (0.80–0.83)	0.90 (0.89–0.92)	<0.001
Healthcare utilization indicator					
Any follow-up visit	502,324 (83.25%)	520,375 (86.25%)	0.97 (0.96–0.97)	1.04 (1.04–1.04)	<0.001
Negative control outcomes					
Follicular cysts of skin	10,917 (1.81%)	11,473 (1.90%)	0.95 (0.93–0.98)	1.16 (1.13–1.19)	<0.001

Data are presented as number of events (%). Hypothyroidism, goiter, and hyperthyroidism were assessed as follow-up thyroid-related diagnostic records rather than incident thyroid outcomes. Follicular cysts of skin were included as a negative-control outcome, and any healthcare visit was assessed as a general healthcare-utilization marker. CI, confidence interval; COVID-19, coronavirus disease 2019; HR, hazard ratio; RR, risk ratio.

All-cause mortality was higher after COVID-19 (22,134 vs 16,822; 3.67% vs 2.79%; HR, 1.70; 95% CI, 1.66–1.73; p<0.001). In contrast, follow-up thyroid-related diagnoses were less frequent, with lower hazards for hypothyroidism, goiter, and hyperthyroidism (HRs, 0.87, 0.87, and 0.90) (**Table 2**). Any follow-up visit occurred in 83.25% of the COVID-19 cohort and 86.25% of controls, although the hazard of a recorded visit was slightly higher after COVID-19 (HR, 1.04; 95% CI, 1.04–1.04). The negative-control outcome showed lower cumulative incidence but a modestly higher hazard (HR, 1.16; 95% CI, 1.13–1.19).

Among patients with at least one corresponding follow-up record, the mean number of thyroid-related diagnostic records was lower in the COVID-19 cohort than in controls for hypothyroidism (7.40 vs. 8.28), goiter (4.08 vs. 4.79), and hyperthyroidism (5.66 vs. 6.66; all p<0.001) (**Table 3**). In contrast, the mean number of all follow-up visits was slightly higher after COVID-19 (56.87 vs. 54.92; p<0.001), whereas the mean number of follicular cyst records—the negative-control outcome—was nearly identical between groups (1.65 vs. 1.68), with the small difference reaching nominal significance only because of the large sample size (p=0.036).

**Table 3:** Frequency of follow-up thyroid-related diagnostic records among patients with at least one corresponding record.

Outcome	COVID-19 group			Control group			
	Patients with outcome	mean ± SD	median	Patients with outcome	mean ± SD	median	*p* value for mean difference
Hypothyroidism	249,181	7.40 ± 7.52	5	287,809	8.28 ± 7.79	6	<0.001
Goiter	59,397	4.08 ± 4.12	3	74,498	4.79 ± 5.32	3	<0.001
Hyperthyroidism	25,912	5.66 ± 7.05	3	31,717	6.66 ± 8.15	3	<0.001
Any follow-up visit	502,324	56.87 ± 69.45	33	520,375	54.92 ± 62.65	34	<0.001
Follicular cysts of skin	10,917	1.65 ± 1.24	1	11,473	1.68 ± 1.35	1	0.036

Values represent the number of corresponding diagnostic records per patient accumulated during the entire follow-up period among patients with at least one corresponding follow-up record. P values were calculated for between-group differences in mean record counts. Medians are presented for descriptive purposes only. COVID-19, coronavirus disease 2019; SD, standard deviation.

### 3.3 Sensitivity analyses

The association between COVID-19 and thyroid cancer was consistent across sensitivity analyses designed to address survival, follow-up intensity, and thyroid-specific ascertainment (**Table 4**). Among patients who survived throughout follow-up, the HR for thyroid cancer was 1.71 (95% CI, 1.57–1.86). Similar estimates were observed when the analysis was restricted to patients with at least one follow-up healthcare encounter (HR, 1.70; 95% CI, 1.56–1.85), patients who underwent thyroid-related testing during follow-up (HR, 1.86; 95% CI, 1.69–2.06), and patients with abnormal TSH values during follow-up (HR, 1.95; 95% CI, 1.73–2.19). These results indicate that the association was not explained solely by differential survival, healthcare retention, or thyroid-related testing.

**Table 4:** Sensitivity analyses for the association between COVID-19 and long-term risk of incident thyroid cancer.

Outcomes	Model I (n=582,404 per group)		Model II (n=502,575 per group)		Model III (n=228,579 per group)		Model IV (n=135,649 per group)	
	HR (95% CI)	*p* value	HR (95% CI)	*p* value	HR (95% CI)	*p* value	HR (95% CI)	*p* value
Thyroid cancer	1.71 (1.57–1.86)	<0.001	1.70 (1.56–1.85)	<0.001	1.86 (1.69–2.06)	<0.001	1.95 (1.73–2.19)	<0.001
Mortality	NA	NA	1.78 (1.75–1.82)	<0.001	1.78 (1.72–1.83)	<0.001	1.91 (1.84–1.98)	<0.001
Hypothyroidism	0.87 (0.86–0.87)	<0.001	0.87 (0.87–0.88)	<0.001	0.92 (0.91–0.92)	<0.001	0.91 (0.91–0.92)	<0.001
Goiter	0.87 (0.86–0.88)	<0.001	0.87 (0.86–0.87)	<0.001	0.94 (0.93–0.96)	<0.001	1.01 (0.98–1.03)	0.596
Hyperthyroidism	0.89 (0.88–0.91)	<0.001	0.90 (0.88–0.91)	<0.001	0.99 (0.97–1.01)	0.256	1.04 (1.01–1.06)	0.003
Any follow-up visit	1.04 (1.03–1.04)	<0.001	1.03 (1.03–1.04)	<0.001	1.10 (1.09–1.10)	<0.001	1.09 (1.08–1.10)	<0.001
Follicular cysts of skin	1.17 (1.14–1.20)	<0.001	1.15 (1.12–1.18)	<0.001	1.16 (1.12–1.20)	<0.001	1.19 (1.14–1.24)	<0.001

Model I included only patients who survived during follow-up. Model II included only patients with at least one healthcare visit during follow-up. Model III was restricted to patients who underwent thyroid-related testing or evaluation during follow-up. Model IV was restricted to patients with an abnormal TSH level (<0.4 or >4 mIU/L) during follow-up. CI, confidence interval; COVID-19, coronavirus disease 2019; HR, hazard ratio; NA, not applicable; TSH, thyroid-stimulating hormone.

Findings were also consistent across strata defined by the type of pre-existing thyroid disease (**Table 5**). The HR for thyroid cancer was 1.66 (95% CI, 1.47–1.86) among patients with baseline hypothyroidism, 1.97 (95% CI, 1.54–2.52) among those with baseline hyperthyroidism, and 1.63 (95% CI, 1.45–1.83) among those with baseline goiter. Mortality remained higher after COVID-19 across all three baseline thyroid-disease strata.

**Table 5:** Sensitivity analyses of the association between COVID-19 and long-term thyroid cancer risk according to type of pre-existing thyroid disease.

Outcomes	Model V (n=427,150 per group)		Model VI (n=49,458 per group)		Model VII (n=121,565 per group)	
	HR (95% CI)	*p* value	HR (95% CI)	*p* value	HR (95% CI)	*p* value
Thyroid cancer	1.66 (1.47–1.86)	<0.001	1.97 (1.54–2.52)	<0.001	1.63 (1.45–1.83)	<0.001
Mortality	1.69 (1.65–1.73)	<0.001	1.79 (1.65–1.94)	<0.001	1.76 (1.67–1.85)	<0.001
Hypothyroidism	0.84 (0.84–0.85)	<0.001	1.18 (1.15–1.21)	<0.001	1.10 (0.98–1.23)	0.098
Goiter	1.04 (1.02–1.06)	<0.001	0.91 (0.88–0.94)	<0.001	1.03 (1.01–1.05)	0.002
Hyperthyroidism	1.13 (1.10–1.16)	<0.001	0.71 (0.70–0.73)	<0.001	0.74 (0.73–0.75)	<0.001
Any follow-up visit	1.03 (1.02–1.03)	<0.001	1.07 (1.06–1.09)	<0.001	0.92 (0.89–0.95)	<0.001
Follicular cysts of skin	1.14 (1.10–1.17)	<0.001	1.23 (1.11–1.36)	<0.001	1.08 (1.07–1.09)	<0.001

Model V included patients with pre-existing hypothyroidism. Model VI included patients with pre-existing hyperthyroidism. Model VII included patients with pre-existing goiter. Data are presented as hazard ratios with 95% confidence intervals. CI, confidence interval; COVID-19, coronavirus disease 2019; HR, hazard ratio.

### 3.4 Subgroup analyses

The association between COVID-19 and thyroid cancer was similar in men and women (**Table 6**). The HR was 1.59 (95% CI, 1.32–1.92) in men and 1.66 (95% CI, 1.52–1.82) in women, with no evidence of effect modification by sex (p for interaction=0.683). Results were also consistent across age groups (**Table 7**). The HR was 1.67 (95% CI, 1.50–1.86) among patients aged 18–65 years and 1.64 (95% CI, 1.44–1.87) among those older than 65 years, with no significant interaction by age (p for interaction=0.834). Mortality showed stronger relative associations in younger patients (p for interaction<0.001).

**Table 6:** Sex-stratified analyses of the association between COVID-19 and long-term risk of incident thyroid cancer and related outcomes.

Outcomes	Male (n=141,655 per group)		Female (n=459,700 per group)		*P* for interaction
	HR (95% CI)	*p* value	HR (95% CI)	*p* value	
Thyroid cancer	1.59 (1.32–1.92)	<0.001	1.66 (1.52–1.82)	<0.001	0.683
Mortality	1.71 (1.65–1.77)	<0.001	1.70 (1.65–1.74)	<0.001	0.794
Hypothyroidism	0.86 (0.85–0.86)	<0.001	0.88 (0.88–0.89)	<0.001	<0.001
Goiter	0.85 (0.82–0.87)	<0.001	0.87 (0.86–0.88)	<0.001	0.145
Hyperthyroidism	0.84 (0.81–0.87)	<0.001	0.91 (0.89–0.93)	<0.001	<0.001
Any follow-up visit	1.01 (1.00–1.02)	0.005	1.05 (1.04–1.05)	<0.001	<0.001
Follicular cysts of skin	1.15 (1.09–1.22)	<0.001	1.17 (1.14–1.21)	<0.001	0.595

Data are presented as hazard ratios with 95% confidence intervals and p values. P values for interaction were calculated to assess whether associations differed by sex. CI, confidence interval; COVID-19, coronavirus disease 2019; HR, hazard ratio.

**Table 7:** Age-stratified analyses of the association between COVID-19 and long-term risk of incident thyroid cancer and related outcomes.

Outcomes	18-65 (n=288,451 for each group)		>65 (n=309,454 for each group)		*P* for interaction
	HR (95% CI)	*p* value	HR (95% CI)	*p* value	
Thyroid cancer	1.67 (1.50–1.86)	<0.001	1.64 (1.44–1.87)	<0.001	0.834
Mortality	1.95 (1.83–2.08)	<0.001	1.66 (1.63–1.70)	<0.001	<0.001
Hypothyroidism	0.89 (0.88–0.90)	<0.001	0.88 (0.87–0.88)	<0.001	0.080
Goiter	0.87 (0.85–0.88)	<0.001	0.89 (0.88–0.90)	<0.001	0.030
Hyperthyroidism	0.88 (0.86–0.90)	<0.001	0.92 (0.90–0.95)	<0.001	0.014
Any follow-up visit	1.09 (1.09–1.10)	<0.001	1.00 (1.00–1.01)	0.152	<0.001
Follicular cysts of skin	1.27 (1.22–1.32)	<0.001	1.14 (1.10–1.18)	<0.001	<0.001

Data are presented as hazard ratios with 95% confidence intervals and p values. P values for interaction were calculated to assess whether associations differed by age group. CI, confidence interval; COVID-19, coronavirus disease 2019; HR, hazard ratio.

### 3.5 Landmark, severity-stratified, competing-risk, and generalizability analyses

The association persisted across alternative landmark definitions (**Table 8**). The HR for thyroid cancer was 1.61 (95% CI, 1.50–1.72) using a 1-year landmark, 1.61 (95% CI, 1.49–1.75) using the primary 2-year landmark (**Table 2**, primary analysis), and 1.93 (95% CI, 1.73–2.16) using a 3-year landmark. In the hospitalization-based severity-stratified analysis (**Table 9**), thyroid cancer risk was similarly increased in non-hospitalized and hospitalized COVID-19 patients (HR, 1.73 and 1.67), with no interaction by hospitalization status (p=0.659). Mortality was higher in hospitalized patients (HR, 2.08 vs. 1.31; p<0.001), supporting the severity classification. The lack of a higher thyroid cancer risk in hospitalized patients suggests the association is not solely driven by hospitalization-related healthcare intensity, although residual surveillance bias cannot be excluded.

**Table 8:** Landmark analyses of the association between COVID-19 and long-term thyroid cancer risk.

Outcomes	1-year		2-year‡		3-year	
	HR (95% CI)	*p* value	HR (95% CI)	*p* value	HR (95% CI)	*p* value
Thyroid cancer	1.61 (1.50–1.72)	<0.001	1.61 (1.49–1.75)	<0.001	1.93 (1.73–2.16)	<0.001
Mortality	1.60 (1.57–1.63)	<0.001	1.70 (1.66–1.73)	<0.001	1.94 (1.89–2.00)	<0.001
Hypothyroidism	0.86 (0.85–0.86)	<0.001	0.87 (0.87–0.88)	<0.001	0.88 (0.88–0.89)	<0.001
Goiter	0.87 (0.86–0.87)	<0.001	0.87 (0.86–0.88)	<0.001	0.87 (0.86–0.88)	<0.001
Hyperthyroidism	0.90 (0.89–0.92)	<0.001	0.90 (0.89–0.92)	<0.001	0.89 (0.87–0.91)	<0.001
Any follow-up visit	0.98 (0.98–0.98)	<0.001	1.04 (1.04–1.04)	<0.001	1.06 (1.06–1.06)	<0.001
Follicular cysts of skin	1.16 (1.13–1.18)	<0.001	1.16 (1.13–1.19)	<0.001	1.18 (1.14–1.22)	<0.001

Data are presented as hazard ratios with 95% confidence intervals and p values. Landmark analyses were performed using 1-, 2-, and 3-year event-free periods after the index date. The 2-year landmark analysis was the primary analysis. CI, confidence interval; COVID-19, coronavirus disease 2019; HR, hazard ratio.

**Table 9:** Severity-stratified analysis of COVID-19 and long-term thyroid cancer risk.

Outcomes	Non-hospitalized COVID-19		Hospitalized COVID-19		*P* for interaction
	HR (95% CI)	*p* value	HR (95% CI)	*p* value	
Thyroid cancer	1.73 (1.57–1.91)	<0.0001	1.67 (1.48–1.89)	<0.001	0.659
Mortality	1.31 (1.27–1.35)	<0.0001	2.08 (2.03–2.13)	<0.001	<0.001
Hypothyroidism	0.91 (0.91–0.92)	<0.0001	0.83 (0.82–0.84)	<0.001	<0.001
Goiter	0.90 (0.89–0.92)	<0.0001	0.81 (0.80–0.82)	<0.001	<0.001
Hyperthyroidism	0.90 (0.88–0.92)	<0.0001	0.89 (0.87–0.91)	<0.001	0.488
Any follow-up visit	1.07 (1.06–1.07)	<0.0001	1.02 (1.01–1.02)	<0.001	<0.001
Follicular cysts of skin	1.23 (1.19–1.27)	<0.0001	1.06 (1.02–1.10)	0.003	<0.001

Data are presented as hazard ratios with 95% confidence intervals. COVID-19 severity was defined according to hospitalization status: non-hospitalized COVID-19 (n=376,209) for each group versus hospitalized COVID-19 (n=278,541 for each group). Each subgroup was compared with separately matched controls without documented SARS-CoV-2 infection. This analysis was exploratory and was not interpreted as a formal dose–response analysis. CI, confidence interval; COVID-19, coronavirus disease 2019; HR, hazard ratio.

**Figure 2:**
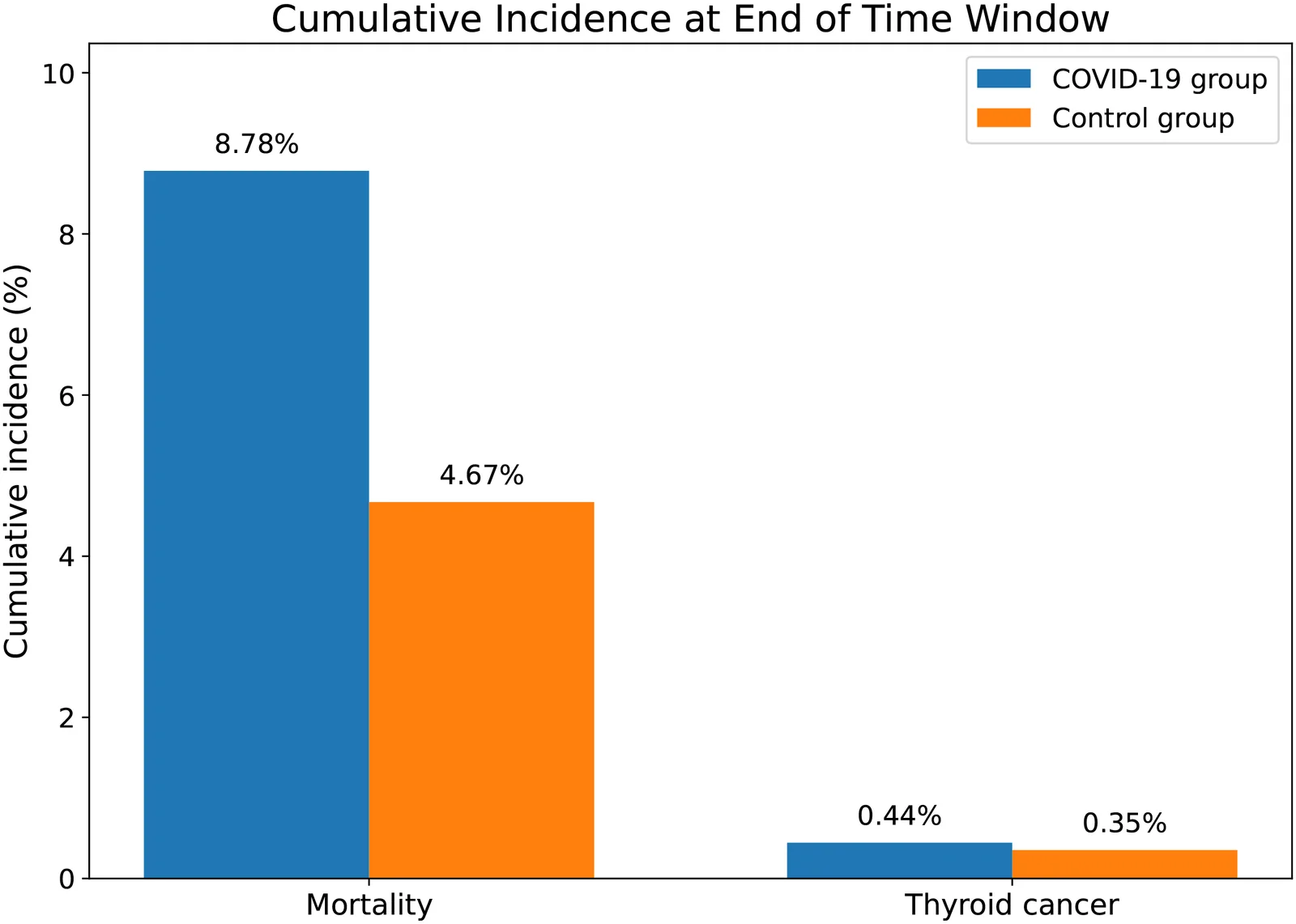
Descriptive competing-risk analysis for incident thyroid cancer with death as a competing event. Aalen–Johansen cumulative incidence curves showing incident thyroid cancer among adults with pre-existing thyroid disease with and without documented COVID-19 in the unmatched cohort. Death was treated as a competing event. This analysis was descriptive and was not used for formal between-group statistical comparison.

In the descriptive competing-risk analysis (**Figure 2**), Aalen–Johansen cumulative incidence curves for thyroid cancer were consistently higher in the COVID-19 cohort than in controls throughout follow-up, with death modeled as a competing event. In the generalizability analysis among adults with type 2 diabetes and no prior thyroid disease (**Table 10**), COVID-19 remained associated with incident thyroid cancer (HR, 1.78; 95% CI, 1.53–2.07); notably, the prespecified positive-control thyroid dysfunction outcomes were all increased after COVID-19 (hypothyroidism, goiter, and hyperthyroidism HRs, 1.39, 1.41, and 1.43), in contrast to the lower hazards observed in the primary thyroid-disease cohort.

**Table 10:** Secondary analysis of COVID-19 and incident thyroid cancer in adults with type 2 diabetes and no prior thyroid disease.

Outcome	COVID-19 group (n=629,370)	Control group (n=629,370)	RR (95% CI)	HR (95% CI)	*p* value
	Events (%)	Events (%)			
Thyroid cancer	412 (0.07%)	308 (0.05%)	1.34 (1.15–1.55)	1.78 (1.53–2.07)	<0.001
Mortality	27,574 (4.38%)	20,713 (3.29%)	1.33 (1.31–1.36)	1.79 (1.76–1.82)	<0.001
Hypothyroidism‡	20,304 (3.23%)	17,845 (2.84%)	1.14 (1.12–1.16)	1.39 (1.36–1.42)	<0.001
Goiter‡	12,344 (1.96%)	11,109 (1.77%)	1.11 (1.08–1.14)	1.41 (1.37–1.44)	<0.001
Hyperthyroidism‡	3,041 (0.48%)	2,648 (0.42%)	1.15 (1.09–1.21)	1.43 (1.35–1.50)	<0.001
Any follow-up visit	487,625 (77.48%)	513,862 (81.65%)	0.95 (0.95–0.95)	1.03 (1.02–1.03)	<0.001
Follicular cysts of skin	8,737 (1.39%)	9,489 (1.51%)	0.92 (0.90–0.95)	1.17 (1.13–1.20)	<0.001

Data are presented as number of events (%) and effect estimates with 95% confidence intervals. CI, confidence interval; COVID-19, coronavirus disease 2019; HR, hazard ratio; RR, risk ratio. ‡Incident hypothyroidism, goiter, and hyperthyroidism were prespecified as positive-control outcomes in current supplemental analysis, given the established association between COVID-19 and new-onset thyroid dysfunction; their detection would indicate adequate sensitivity of the analytic approach to identify true post-COVID thyroid effects.

## 4 Discussion

In this large, multinational cohort of adults with pre-existing thyroid disorders, SARS-CoV-2 infection was linked to a delayed rise in newly diagnosed thyroid cancer, even after applying propensity score matching and a 2-year landmark approach. This relationship remained consistent across multiple analytical strategies, including sensitivity, subgroup, landmark, hospitalization-based severity-stratified, and competing-risk analyses, and was reproduced in a secondary analysis of patients with type 2 diabetes and no prior thyroid disease. These findings argue against measured healthcare utilization as the sole explanation, although residual surveillance bias cannot be excluded. Nonetheless, considering the well-recognized tendency for thyroid cancer to be overdiagnosed, these findings should be viewed as demonstrating a consistent epidemiologic signal rather than definitive evidence of new cancer development.

Our findings differ from and extend prior TriNetX-based evidence on COVID-19 and thyroid cancer because the present study was specifically designed to address several methodological limitations and sources of bias that complicated earlier interpretation. Wang et al. (31) reported a modest association in the general adult population (HR approximately 1.10). By contrast, our analysis focused on adults with established thyroid disease, a population already engaged in thyroid surveillance and at elevated baseline risk for thyroid cancer. The present design also incorporated more extensive baseline covariate adjustment, avoided stratification according to post-exposure thyroid dysfunction, and included a prespecified landmark design, negative-control and healthcare-utilization analyses, multiple surveillance-focused sensitivity analyses, hospitalization-based severity stratification, a descriptive competing-risk analysis, and a secondary generalizability cohort. In this setting, we observed a higher estimate (HR, 1.61; 95% CI, 1.49–1.75), suggesting a more apparent association in a high-risk population, although differences in study design and detection context should be considered. Restriction to patients with pre-existing thyroid disease may have reduced heterogeneity in detection opportunity between groups, while the 2-year landmark design further excluded cancers diagnosed during early post-infectious evaluation.

The incidence of thyroid cancer is strongly influenced by diagnostic intensity and incidental detection of small papillary tumors (33, 37). Because COVID-19 may increase healthcare contact during recovery, surveillance-driven diagnosis could mimic an increased cancer risk. To better interpret the observed association between COVID-19 and thyroid cancer risk, two key aspects of our study design warrant consideration. First, restricting the primary cohort to patients with pre-existing thyroid disorders placed both groups within a clinical setting in which thyroid function testing, neck imaging, and endocrine follow-up are already common. This reduced, although did not eliminate, imbalance in opportunities to detect occult thyroid cancer. Second, the 2-year landmark excluded cancers recorded during the period most susceptible to acute post-COVID evaluation, making the analysis more relevant to delayed events. The persistence of the association after restricting the cohort to patients with follow-up healthcare encounters, thyroid-related testing, and abnormal TSH values further argues against a simple ascertainment explanation. However, these analyses cannot definitively distinguish between true biological effects and earlier detection of previously undiagnosed subclinical cancers. Accordingly, the findings are consistent with a delayed increase in thyroid cancer diagnoses following COVID-19, but they should be interpreted with caution given the high diagnostic sensitivity for thyroid malignancies.

Globally, thyroid cancer was the seventh most commonly diagnosed cancer in 2022, with more than 821,000 new cases and an age-standardized incidence rate of 9.1 per 100,000 person-years (38). In our matched cohort of patients with pre-existing thyroid disease, the post-landmark event proportions were 0.23% after COVID-19 and 0.18% among controls, corresponding to approximately 227 and 175 events per 100,000 patients, respectively. These absolute proportions cannot be directly compared with annual age-standardized population incidence rates because our cohort was clinically enriched for thyroid disorders and patients had variable follow-up durations of up to 4 years after the landmark. Nevertheless, they demonstrate that the absolute frequency of newly recorded thyroid cancer remained low despite the observed relative association.

The negative-control analysis provided partial reassurance, but not definitive exclusion, of nonspecific detection bias because follicular cysts of skin showed no increase in cumulative incidence but a modestly higher hazard after COVID-19. Overall follow-up contact was also not substantially greater in the COVID-19 group, and thyroid-related diagnostic records for hypothyroidism, goiter, and hyperthyroidism were generally less frequent after infection. Importantly, the association with thyroid cancer persisted, and was numerically higher, when analyses were restricted to patients with follow-up healthcare encounters, thyroid-related testing, or abnormal TSH values. These sensitivity analyses improved comparability in observed clinical follow-up, and the association with thyroid cancer remained evident. This finding supports the robustness of the association and argues against differential healthcare retention or thyroid-related testing as the sole explanation, although residual surveillance bias and selection effects cannot be excluded. The estimate was stable across the 1- and 2-year landmarks and was higher after the 3-year landmark, making short-term post-infectious diagnostic activity an unlikely sole explanation. Although residual surveillance bias cannot be excluded, the consistency of these findings suggests that differential detection alone may not account for the delayed increase in recorded thyroid cancer after COVID-19.

Analysis stratified by hospitalization status offered additional insight. The increase in thyroid cancer risk was comparable between non-hospitalized and hospitalized COVID-19 patients, with no significant interaction by hospitalization status. By contrast, mortality was markedly higher among hospitalized patients, supporting hospitalization status as a clinically meaningful severity marker. Because hospitalized patients may have greater healthcare contact and diagnostic evaluation, a stronger thyroid cancer signal in this group would be expected if the association were mainly driven by hospitalization-related surveillance. However, thyroid cancer risk was not higher among hospitalized patients, suggesting that the observed association is unlikely to be explained solely by increased healthcare contact related to follow-up intensity.

If the association is biological, tumor promotion or accelerated recognition of pre-existing subclinical lesions may be more plausible than *de novo* carcinogenesis within this follow-up window. Thyroid follicular cells are known to express ACE2, and SARS-CoV-2 RNA has been identified in thyroid tissue (20, 39). Infection-related processes, such as inflammation, oxidative stress, immune imbalance, and disruption of tumor-suppressor pathways, may create conditions that favor tumor promotion or progression (40, 41). This interpretation is consistent with emerging evidence from other malignancies: experimental models have shown that respiratory viral infections, including SARS-CoV-2, can induce interleukin-6-dependent reactivation and expansion of dormant breast cancer cells, while human observational data have linked COVID-19 to cancer-related mortality and pulmonary metastatic progression among cancer survivors (42). Large population-based cohort studies have also reported increased risks of several autoimmune and chronic inflammatory disorders after COVID-19, including rheumatoid arthritis, psoriasis, spondyloarthritis, Graves’ disease, and vasculitis (43, 44). Together, these observations support, but do not prove, a broader post-infectious framework involving persistent cytokine signaling, altered immune surveillance, and loss of immune tolerance. These mechanisms could be particularly relevant in individuals with existing thyroid disorders, where chronic inflammation, autoimmune activity, or structural abnormalities are already present. Given the relatively short follow-up period, it is improbable that COVID-19 directly caused new differentiated thyroid cancers to develop and become clinically apparent. Instead, it is more plausible that SARS-CoV-2 infection may hasten the growth or detection of previously undiagnosed, subclinical lesions in individuals with underlying thyroid vulnerability. However, this interpretation remains speculative.

The association with incident thyroid cancer was reproduced in a separate population of adults with type 2 diabetes but no documented pre-existing thyroid disease. Although this secondary analysis was conducted within the same data network and should not be considered external validation, it suggests that the observed association was not limited to the primary thyroid-disease cohort. Notably, patterns of nonmalignant thyroid-related diagnoses differed between the primary thyroid-disease cohort and the secondary diabetes cohort, whereas the association with thyroid cancer was observed in both populations. This discordance suggests that the thyroid cancer signal did not simply mirror a uniform increase in thyroid-related diagnostic recording after COVID-19.

The principal clinical implication is that these findings do not justify routine thyroid cancer screening or additional thyroid imaging based solely on a history of COVID-19. Rather, the findings support heightened clinical vigilance and adherence to established thyroid follow-up among patients with pre-existing thyroid disorders after COVID-19. New thyroid nodules, persistent neck symptoms, cervical lymphadenopathy, or unexplained changes in thyroid function should prompt timely clinical evaluation. The observed increase in absolute risk is modest, and clinicians should be cautious to avoid unnecessary ultrasound examinations or biopsies in asymptomatic individuals, given the known issue of thyroid cancer overdiagnosis. Thus, prior COVID-19 may be considered as part of the broader clinical context, but it should not independently trigger intensified thyroid cancer screening. Consistent with current active surveillance strategies, appropriately selected patients with known or suspected intrathyroidal low-risk differentiated thyroid cancer may be followed with serial imaging as an alternative to immediate surgery, with surgery considered if disease progression is suspected or according to patient preference (45). A history of COVID-19 alone should not modify these established diagnostic or management pathways.

Several limitations should be acknowledged. First, TriNetX does not provide detailed tumor characteristics, including histology, tumor size, stage, or mode of detection; therefore, we could not determine whether the excess thyroid cancers were clinically significant tumors or predominantly small incidental papillary lesions. Second, COVID-19 exposure was defined using diagnostic codes and positive laboratory results, which may have missed asymptomatic, undocumented, or home-tested infections. Such misclassification would likely attenuate the observed association. Third, laboratory and imaging data were incomplete, limiting detailed assessment of thyroid function, antibody status, ultrasound findings, and nodule characteristics. In addition, fine-needle aspiration biopsy, thyroidectomy, radioactive iodine treatment, and other low-frequency thyroid-directed procedures or treatment modalities were not incorporated into the matching model. We therefore could not fully compare or control for differences in detailed thyroid diagnostic and treatment intensity, which may have influenced the likelihood or timing of thyroid cancer detection. Fourth, although we used propensity score matching, landmark analysis, healthcare-utilization measures, and negative-control outcomes, residual confounding and unmeasured differences in surveillance intensity cannot be excluded. Finally, limited generalizability is a major limitation. Because both cohorts were derived from the same TriNetX network and included selected high-risk populations, the findings may not apply to the general population or healthcare settings with different thyroid screening practices. External validation is required.

## 5 Conclusion

In this large propensity score–matched cohort of adults with pre-existing thyroid disease, COVID-19 was associated with a delayed increase in incident thyroid cancer after a 2-year landmark period. The association was consistent across multiple analyses and was not readily explained by measured healthcare utilization alone. However, the negative-control findings indicate that residual detection bias remains possible. These findings indicate a possible epidemiologic signal, but they should be interpreted with caution and do not establish a causal relationship or direct carcinogenesis. The excess risk may reflect biological promotion, accelerated recognition of subclinical disease, or both. Further studies with tumor stage, histology, ultrasound findings, and prospective surveillance data are needed to clarify clinical relevance and mechanism.

## Data Availability

The datasets generated and/or analyzed during the current study are not publicly available and cannot be shared directly by the corresponding author because they were obtained from the TriNetX Global Collaborative Network under data-use restrictions. The TriNetX platform provides only aggregated, de-identified results and does not permit authors to export or redistribute patient-level data. Aggregate results and query/code definitions may be made available from the corresponding author upon reasonable request and with permission from TriNetX.
