# Supplementary material for: Long-term risk of incident thyroid cancer after COVID-19 in patients with thyroid disorder: a multicenter propensity score–matched cohort study
Source: Front Endocrinol (Lausanne). 2026 Aug 11;17:1926365. doi: 10.3389/fendo.2026.1926365 (PMC13503246; doi:10.3389/fendo.2026.1926365)
Supplement: Supplementary file 1 [file Table1.docx]

**Supplemental Table 1. Codes used to define cohort eligibility, exposure, and exclusion criteria**

| Criterion | Code set / definition |
| --- | --- |
| Age at index | TriNetX demographic variable; age ≥18 years |
| Pre-existing thyroid disease | ICD-10-CM E00–E07 |
| COVID-19 diagnosis | ICD-10-CM U07.1 |
| SARS-CoV-2 RNA positivity | TriNetX laboratory term TNX:9088, SARS coronavirus 2 and related RNA [Presence] |
| Control cohort without documented SARS-CoV-2 infection | Absence of ICD-10-CM U07.1 and absence of TriNetX laboratory term TNX:9088 |
| Healthcare encounter during the 5-year baseline period | TriNetX visit term TNX |
| Pre-existing or early thyroid cancer before the start of outcome assessment | ICD-10-CM C73, malignant neoplasm of thyroid gland, before index or within 24 months after index |
| Early death or uncertain mortality coding during the 24-month landmark period | TriNetX deceased status; ICD-10-CM R99 |
| Advanced chronic kidney disease | ICD-10-CM N18.4, N18.5 |
| End-stage renal disease or dialysis dependence | ICD-10-CM N18.6, Z99.2 |
| Type 1 diabetes mellitus | ICD-10-CM E10 |
| Gestational diabetes mellitus | ICD-10-CM O24.4 |
| Systemic connective tissue disorders | ICD-10-CM M30–M36 |
| Malignant neoplasm of head, face, and neck | ICD-10-CM C76.0 |
| Malignant neoplasms of lip, oral cavity, and pharynx | ICD-10-CM C00–C14 |
| Malignant neoplasm of nasal cavity and middle ear | ICD-10-CM C30 |
| Malignant neoplasm of accessory sinuses | ICD-10-CM C31 |
| Malignant neoplasm of larynx | ICD-10-CM C32 |
| Benign neoplasm of thyroid gland | ICD-10-CM D34 |
| Carcinoma in situ of thyroid and other endocrine glands | ICD-10-CM D09.3 |
| Personal history of irradiation | ICD-10-CM Z92.3 |
| Family history of primary malignant neoplasm | ICD-10-CM Z80 |

**Supplemental Table 2. Codes for variables used in propensity score matching**

| Matching variable | Code set / definition |
| --- | --- |
| Age at index | TriNetX demographic variable |
| Sex | TriNetX demographic variable |
| Body mass index >30 kg/m² | TriNetX laboratory/vital term 9083; BMI >30 kg/m² |
| White | TriNetX race variable |
| Black or African American | TriNetX race variable |
| Asian | TriNetX race variable |
| Other hypothyroidism | ICD-10-CM E03 |
| Essential hypertension | ICD-10-CM I10 |
| Disorders of lipoprotein metabolism and other lipidemias | ICD-10-CM E78 |
| Neoplasms | ICD-10-CM C00–D49 |
| Overweight and obesity | ICD-10-CM E66 |
| Diabetes mellitus | ICD-10-CM E08–E13 |
| Nontoxic goiter | ICD-10-CM E04 |
| Ischemic heart diseases | ICD-10-CM I20–I25 |
| Nicotine dependence | ICD-10-CM F17 |
| Cerebrovascular diseases | ICD-10-CM I60–I69 |
| Diseases of liver | ICD-10-CM K70–K77 |
| Other disorders of thyroid | ICD-10-CM E07 |
| Chronic kidney disease | ICD-10-CM N18 |
| Thyrotoxicosis [hyperthyroidism] | ICD-10-CM E05 |
| Other chronic obstructive pulmonary disease | ICD-10-CM J44 |
| Heart failure | ICD-10-CM I50 |
| Alcohol-related disorders | ICD-10-CM F10 |
| Malnutrition | ICD-10-CM E40–E46 |
| Glucocorticoids | ATC H02AB |
| Thyroid hormones | ATC H03AA |
| Antilipemic agents | ATC C10 / TriNetX medication class CV350 |
| COVID-19 vaccination | CVX 213 / TriNetX SARS-CoV-2 vaccine term |
| Insulins and analogues | ATC A10A |
| Glucagon-like peptide-1 analogues | ATC A10BJ |
| Antithyroid preparations | ATC H03B |
| Sodium-glucose cotransporter-2 inhibitors | ATC A10BK |
| Estimated glomerular filtration rate ≥60 mL/min/1.73 m² | TriNetX laboratory term 8001; eGFR ≥60 mL/min/1.73 m² |
| Hemoglobin ≥12 g/dL | TriNetX laboratory term 9014; hemoglobin ≥12 g/dL |
| Albumin ≥3.5 g/dL | TriNetX laboratory term 9045; albumin ≥3.5 g/dL |
| Hemoglobin A1c ≥9% | TriNetX laboratory term 9037; HbA1c ≥9% |
| Thyrotropin within reference range | TriNetX laboratory term 9040; TSH 0.4–4 mIU/L |
| Thyroid-stimulating hormone testing | CPT 84443 |
| Diagnostic ultrasound procedures of the head and neck | TriNetX procedure term 1010760 |
| Ultrasound of soft tissues of the head and neck | CPT 76536 |
